# Graphene: A Versatile Carbon-Based Material for Bone Tissue Engineering

**DOI:** 10.1155/2015/804213

**Published:** 2015-06-01

**Authors:** Nileshkumar Dubey, Ricardo Bentini, Intekhab Islam, Tong Cao, Antonio Helio Castro Neto, Vinicius Rosa

**Affiliations:** ^1^Discipline of Oral Sciences, Faculty of Dentistry, National University of Singapore, Singapore 119083; ^2^Centre for Advanced 2D Materials and Graphene Research Centre, National University of Singapore, Singapore 117542; ^3^Discipline of Oral and Maxillofacial Surgery, Faculty of Dentistry, National University of Singapore, Singapore 119083

## Abstract

The development of materials and strategies that can influence stem cell attachment, proliferation, and differentiation towards osteoblasts is of high interest to promote faster healing and reconstructions of large bone defects. Graphene and its derivatives (graphene oxide and reduced graphene oxide) have received increasing attention for biomedical applications as they present remarkable properties such as high surface area, high mechanical strength, and ease of functionalization. These biocompatible carbon-based materials can induce and sustain stem cell growth and differentiation into various lineages. Furthermore, graphene has the ability to promote and enhance osteogenic differentiation making it an interesting material for bone regeneration research. This paper will review the important advances in the ability of graphene and its related forms to induce stem cells differentiation into osteogenic lineages.

## 1. Introduction

Bone tissue regeneration is of high interest to promote faster healing and reconstruction of large bone defects created by tumor resection, skeletal abnormalities, fractures, and infection. The development of this field requires the use of substrates that enable cell attachment, proliferation, and differentiation [1–3]. Several different materials can initiate, stimulate, and sustain the series of complex events that lead to cell differentiation and osteogenesis [3, 4]. For example, collagen can offer suitable surface chemistry for cell growth and differentiation [5, 6] but possesses poor mechanical properties and is prone to immune response [3, 6, 7]. Hydrogels with tunable physical and chemical properties may positively direct stem cell fate [6, 8]. However, their limitations may include lack of cell-specific bioactivities and it is challenging to create large structures due to the need of a highly cross-linked network that can interfere in cell behavior [4, 6, 8, 9]. Therefore, materials with intrinsic characteristics that can sustain cell growth and induce differentiation possess a great potential for stem cell research.

Graphene is a single atomic sheet of conjugated sp^2^ carbon atoms and is the thinnest, lightest, and maybe the strongest material known [10, 11]. Its electrical conductivity and charge carrier mobility surpass the most conductive polymers by several orders [12] making graphene a revolutionary material for electronic devices such as batteries, semiconductors, electrochemical sensors, and others. As it can be easily functionalized, graphene has also opened avenues for use in biomedical applications (e.g., biosensors, nanocarriers for drug and gene delivery, and devices for cell imaging and phototherapy for cancer) [12–17]. As graphene can be synthesized in a relatively pure form and offers tunable surface, it has emerged as an interesting substrate for experiments with anchorage-dependent cells such as mesenchymal stem cells (MSCs), neuronal stem cells, induced pluripotent stem cells, and others [12, 15, 18–23].

## 2. Graphene and Its Unique Properties

Since its discovery in 2004 by Nobel laureates Geim and Novoselov [24], graphene has attracted massive interest because of its unique physical, mechanical, and chemical properties [10–12, 25–27, 29–32]. 

*Graphene Physical, Chemical, and Mechanical Properties*

- Thinnest, strongest, and stiffest imaginable material [24].
- Almost transparent [25, 26].
- Most stretchable crystal (20% elasticity) [24].
- Recording thermal conductivity [24].
- Highest current density at room temperature [26].
- Completely impermeable [27].
- Highest intrinsic mobility (100 times more than in Si) [26].
- Conducting electricity in the limit of no electrons [26].
- Large surface area (~2600 m^2^ g^−1^) [28].
- Longest mean free path at room temperature (micron range) [26].

Due to its unique structure and a pure aromatic carbon system [29], it has high electron mobility at room temperature [26]. It also has exceptional thermal and chemical stability and can work as an impermeable barrier supporting pressure differences larger than one atmosphere [24, 27]. Though it is almost transparent, it absorbs approximately 2.3% of white light which makes it slightly visible to the naked eye [25]. Moreover, it is flexible and can adapt and deform in the direction normal to its surface. The large surface area, close to 2600 m^2^ g^−1^, makes it an attractive platform for anchorage of large amounts of molecules [28, 33].

**Figure 1:**
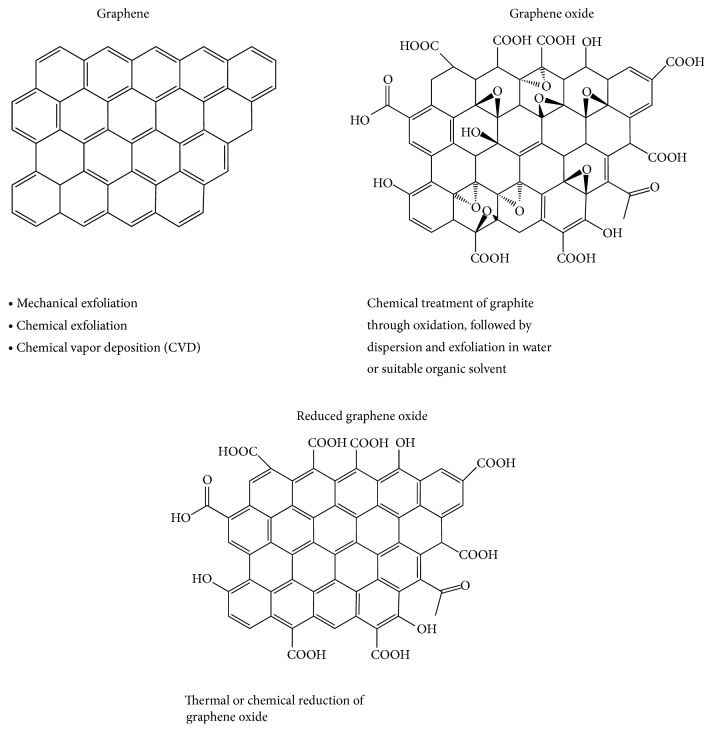
Methods of obtaining graphene and its derivatives.

Graphene-related materials can be classified based on either number of layers (e.g., mono- or multilayered graphene) or their chemical modification such as graphene oxide (GO) or reduced GO (rGO) (Figure 1). GO is a highly oxidized form of graphene prepared by oxidation of graphite. This amphiphile compound allows surface functionalization and can be dispersed in aqueous solution, making it an attractive candidate for gene and drug delivery and substrate modification [12, 16, 17, 34–37]. rGO can be further reduced to graphene-like sheets by removing the oxygen-containing groups with the recovery of a conjugated structure [12, 32].

Graphene in different forms can be obtained using “top-down” and “bottom-up” methods. The “top-down” approaches include mechanical and/or chemical exfoliation of graphite. The mechanical method, also known as the “Scotch tape” or peel-off technique, allows the detachment of micrometer-sized graphene flakes from a graphite crystal using adhesive tape [24, 32, 38]. In the chemical exfoliation, graphite is oxidized using strong acids such as sulphuric or nitric acid. This procedure inserts oxygen atoms between individual graphene sheets and forces them apart, resulting in a suspension of GO sheets that can be filtered to isolate GO flakes [30, 39]. As GO presents oxygen functionalities it can be well dispersed in water, physiological media, and other organic solvents [40].

Graphene can also be obtained using “bottom-up” approaches like epitaxial growth and chemical vapor deposition (CVD) [38, 41]. The latter is a versatile and scalable method for production of large scale and high quality graphene that can be transferred to various substrates [42]. In typical CVD process, a copper or nickel substrate is annealed and precursor gases (CH_4_ and H_2_) are pumped into a reaction chamber (usually a quartz tube) at high temperatures (~1000°C). The high temperature leads to the pyrolysis of precursors and dissociates carbon atoms which then react with the substrate to produce the thin film of graphene [38]. CVD-grown graphene is flexible and hydrophobic that can be used as a substrate to promote cell proliferation and enhance some of their functions [12, 33, 35, 43–47].

**Figure 2:**
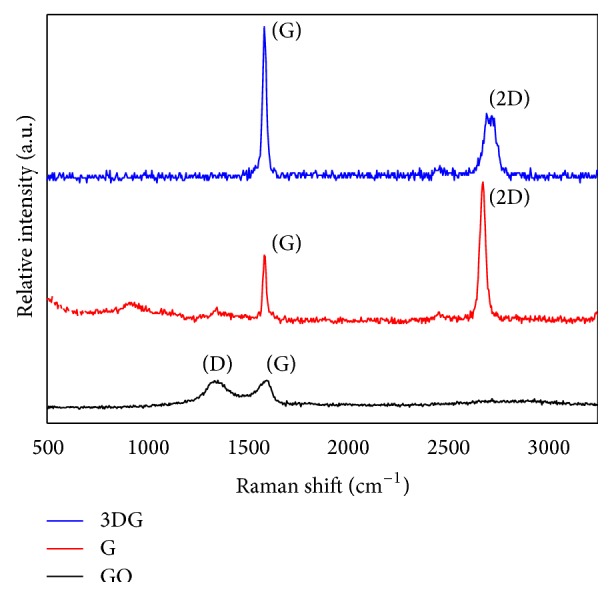
Characterization of graphene, graphene oxide, and three-dimensional graphene-based scaffold by Raman spectroscopy.

The different methods to produce graphene result in materials with different number of layers and/or chemical groups. Raman fingerprints for different groups and number of layers reflect changes in the electron bands and allow unmistakable identification and characterization of graphene by the analysis of three peaks: G, 2D, and D (Figure 2). The G and 2D bands are the most prominent in graphene samples: the G-band (~1587 cm^−1^) arises from the stretching of the C–C bond in graphitic materials whereas the D band (~1340 cm^−1^) is only activated if disorder or defects are present. The 2D band (2500–2800 cm^−1^) is present in all types of sp^2^ carbon materials and is used to determine the number of layers of graphene [48].

## 3. Cytotoxicity and Biocompatibility of Graphene

Graphene and its derivatives are interesting materials for biomedical applications since carbon is the basis of organic chemistry [24, 34]. However, the shape and physical and chemical characteristics of carbonaceous nanomaterials play an extremely important role in how they interact with cells, tissues, and organs [49].

Anchorage-dependent cells need to adhere to substrates in order to spread, proliferate, and perform their functions [3, 14, 46, 47, 50, 51]. The CVD-grown graphene allows human MSCs attachment and proliferation similar to other substrates used for cell culture [33, 46, 47, 52]. Bone marrow-derived MSCs from goats are also capable of proliferating in culture plates coated with GO (0.1 mg/mL) [37].

Although substrates coated with graphene-based materials are not cytotoxic, the use of the material in solutions might pose hazards to cells and tissues. Cell viability may decrease significantly in solutions with high concentrations of pristine graphene (50 *μ*g/mL) as it accumulates on the cell membrane leading to high levels of oxidative stress [53]. Graphene microsheets with lateral dimensions lower than 5 *μ*m can enter mammalian cells initiated by spontaneous penetration of lipid bilayers in a dominant edge-first or corner-first mode. Nonetheless, the uptake of microsheets larger than 5 *μ*m in lateral dimensions is often incomplete [54]. When PC12 cells, a pheochromocytoma of the rat adrenal medulla, are exposed to graphene sheets in solution (0.1 *μ*g/mL), there is an increase in reactive oxygen species production and decrease in metabolic activity. However, at concentrations of 0.01 and 0.1 *μ*g/mL, there is no increase in lactate dehydrogenase, an enzyme released upon membrane damage [49].

Carbon-based materials present different effects when administered* in vivo* as they present diverse patterns of biodistribution [55, 56]. Mice injected with graphene nanosheets exhibited a Th2 immune response in the lung, whereas those injected with multiwalled carbon nanotubes (CNT) presented it in the spleen. The pulmonary instillation of multiwalled CNT in mice induces IL-33 production and may function as an alarm in response to nanomaterial exposure [56].

One strategy used to improve graphene's biocompatibility relies on the generation of covalent bonding of polyethylene glycol (PEG) to minimize oxidation. There was no considerable toxicity after injecting mice with 20 mg/kg with PEGylated graphene as evidenced by histological and hematological analysis after 90 days. In fact, the graphene sheets levels in most organs were very low after three days from the injection. The relatively slow but persistent decrease of the material concentration in the liver and spleen suggests that the clearance of graphene nanosheets from the mouse body happens through both renal and fecal excretions [55].

As graphene-based materials can be functionalized, there is an increased interest in using them for biomedical applications. In fact, the surface functionalization may be an important step for pacifying its strong hydrophobicity that may be associated with toxic effects. Nonetheless, the potential long-term adverse effects of functionalized graphene cannot be neglected [53]. Further studies regarding the safety, biodistribution, and adverse effects are needed before the material can be used at large in biological systems.

## 4. Graphene's Capability to Induce and Improve Stem Cell Osteogenic Differentiation

Major bone reconstruction represents a major challenge and is a global health problem. Stem cell-based therapy might be a promising solution but it requires the constant development of biocompatible platforms that can promote and enhance cell viability, attachment, migration, and differentiation [1, 3]. Several materials such as poly-L-lactic acid (PLLA), polycaprolactone (PCL), chitosan, and composites based on these materials are constantly developed and improved to match some properties of native bone [3, 57, 58]. However, fine-tuning the mechanical properties and chemical and physical characteristics to match native bone properties is rather challenging [58]. In some polymers, such as PLLA and PCL, the lack of sites for cell adhesion may require chemical modification to provide such cues to allow stem cell adhesion. Furthermore, their byproducts upon degradation can trigger immune responses [59]. Bioactive inorganic materials are also widely used in bone research. However, due to their brittle nature, they often fail to match the fracture toughness of bone and may not be suitable for load bearing applications [60].

Graphene has emerged as a promising material for stem cell research due to its unique mechanical, physical, and chemical properties (Table 1) [20, 24, 61]. Graphene-based materials allow stem cell attachment and growth and enhance osteogenic differentiation supporting its introduction as an alternative material for bone regeneration research [14, 21, 23, 33, 45–47, 52].

Cell adhesion, viability, and proliferation rate are directly related to the biocompatibility of the substrate [3, 6, 51]. In fact, cell attachment and differentiation are greatly affected by the surface characteristics of materials and by forces generated at the cell/material interfaces. [46, 61, 62]. Graphene-based coatings are noncytotoxic and allow the attachment and proliferation of fibroblasts, osteoblasts, and mesenchymal stem cells (MSC) and have been shown to enhance stem cell differentiation [33, 43, 45–47, 62–64]. Human osteoblast-like cells (SAOS-2) and MSC seeded on CVD graphene presented higher proliferation than silicon dioxide (SiO_2_) after 48 hours of incubation. Furthermore, MSCs on graphene assume a spindle shaped morphology whereas those cultured on SiO_2_ substrates form separate islands of polygonal cells [46]. As cell morphology is related to stem cell commitment to different lineages [65] the spindle shaped cell structure of MSC on graphene may possess higher potential for osteoblast differentiation as compared to the SiO_2_ substrate [46]. Furthermore, cells cultured on CVD graphene present higher proliferation in comparison to SiO_2_, graphene oxide, and polydimethylsiloxane (PDMS) substrates [33, 46] but similar to those cultured on glass, one of the most used substrates for cell culture [47].

**Figure 3:**
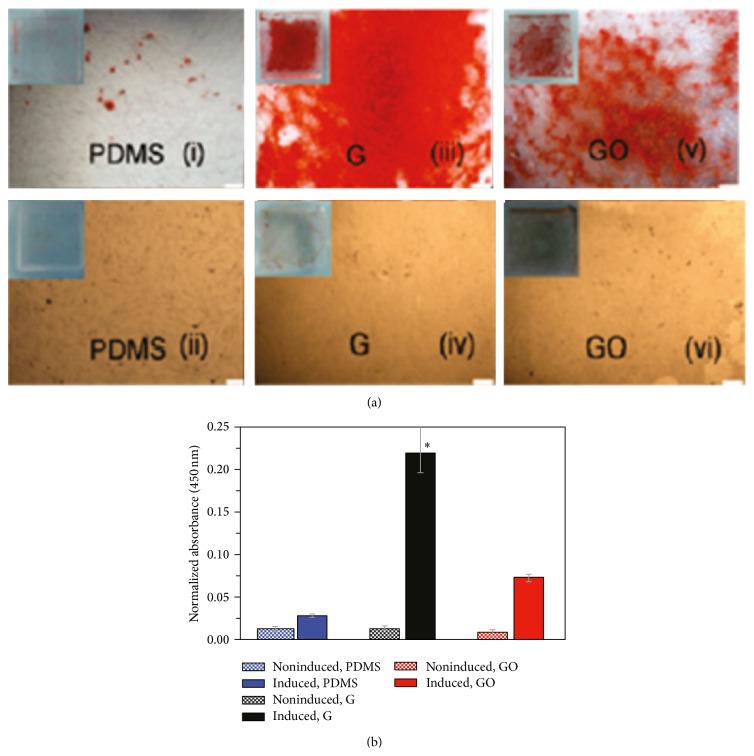
(a) Alizarin Red staining after 12 days of incubation of BMSC on PDMS, graphene (G), and GO with ((i), (ii), and (iv)) and without osteogenic medium ((ii), (iv), and (vi)). (b) Quantification demonstrated a significantly higher amount of Alizarin Red staining in the MSCs differentiated on graphene. Reprinted with permission from [33] (Copyright (2011) American Chemical Society).

**Figure 4:**
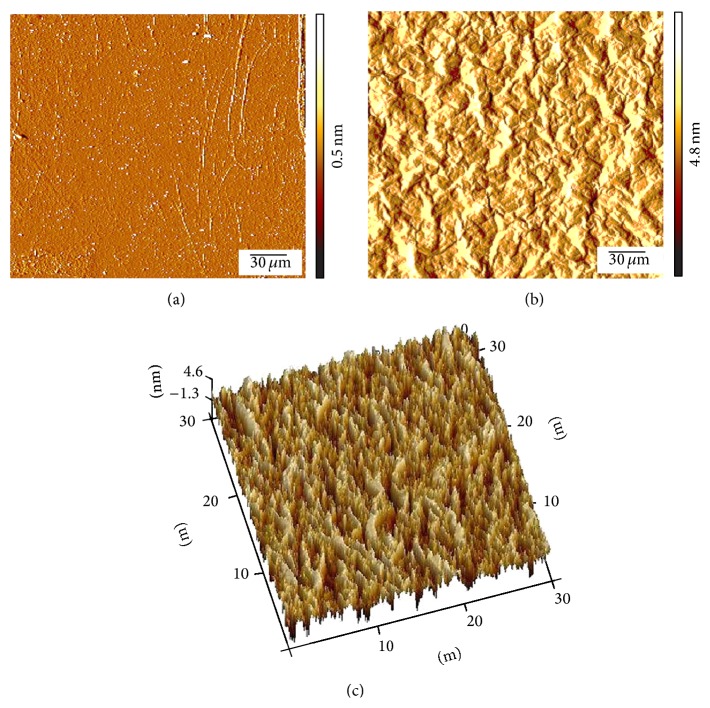
Characterization by atomic force microscopy of (a) CVD-grown graphene; (b) and (c) GO (10 mg/mL).

Although cell proliferation is not improved by pristine graphene, the material enhances stem cell differentiation towards osteoblastic lineage [33, 45, 47, 52]. MSCs exhibit accelerated osteogenic differentiation when cultured on two-dimensional graphene sheets as compared to GO and PDMS in the presence of an osteogenic induction medium (Figure 3) [33, 47]. Self-supporting graphene hydrogel film induced higher levels of osteogenic differentiation of rat bone marrow stem cells (BMSC) in growth medium [66]. MSC cultured on graphene oxide nanoribbon (GONR) and reduced GONR (rGONR) grids showed 3.4- and 2.7-fold increase in the mineralized deposition than those cultured on PDMS and glass [45]. When glass and Si/SiO_2_ were coated with CVD graphene, MSCs present high expression of osteocalcin (OCN) as compared to the uncoated materials [47]. OCN is identified as a late bone marker in osteoblasts [67]. These increments in differentiation might be attributed to the high modulus of elasticity and stiffness of graphene [47]. Furthermore, graphene can sustain lateral stress that may influence cytoskeletal tension leading to changes in cytoskeleton organization and structures which influence cell differentiation [47, 68]. It is known that soft matrices that mimic brain are neurogenic, stiffer substrates that mimic muscle are myogenic, and comparatively rigid matrices similar to collagenous bone induce osteogenic differentiation [69]. Other factors for the increased differentiation may be attributed to the presence of wrinkles and ripples on graphene [33, 45, 47]. These are created during the production of graphene. The CVD graphene is usually synthesized at high-temperatures (~1000°C) and it experiences negative thermal expansion while cooling. Thus, graphene expands laterally while the metal used as the sacrificial substrate shrinks, resulting in the formation of those wrinkles and ripples. Examples of superficial characteristics for both CVD-grown graphene and GO are presented in Figure 4 [34].

**Figure 5:**
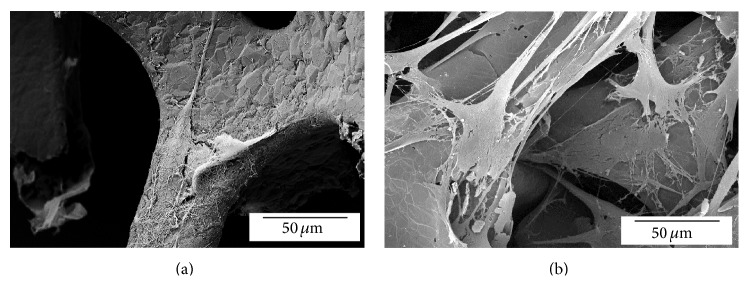
Periodontal ligament stem cell in 3DGP after 3 (a) and 5 days (b) on culture. Cells are able to attach and proliferate into the three-dimensional surface of the graphene-based scaffold.

It is known that the transport phenomena of cytokines, chemokines, and growth factors are drastically different between two- and three-dimensional (3D) microenvironments interfering in signaling transduction, cell-cell communications, and tissue development [3, 6, 70]. Graphene 3D construct (3DGp) can be synthesized via CVD using a nickel foam as template and are capable of inducing spontaneous neuronal and osteogenic differentiation of MSC [52, 64]. Cells in 3DGp presented a spindle shaped and elongated morphology with thin and aligned nuclei, typical of osteoprogenitor cells and expressed osteogenic markers OCN and osteopontin (OPN) even without the use of osteogenic medium [52]. Recently, our group has succeeded in culturing periodontal ligament stem cells (PDLSC) in 3DGp (Figure 5). After 5 days, the surface of 3DGp was covered by cells having an elongated shape, showing that 3DGp is a suitable substrate for PDLSC attachment and proliferation.

Although graphene holds the potential to induce spontaneous osteogenic differentiation of stem cells, this property is significantly enhanced by the use of chemical inductors for osteogenic differentiation [33, 45, 47, 52]. When MSCs were cultured on CVD graphene with osteogenic medium, the extent of mineralized deposition was remarkably higher than that observed for PDMS and GO sheets [33]. Similarly, CVD graphene substrate was able to induce osteogenic differentiation of MSC at the same rate as Si/SiO_2_ substrates treated with BMP-2 [47]. The extent of the induced differentiation of MSC cultured on GONR and rGONR grids was 6.4- and 16.3-fold higher than those obtained for PDMS substrates [45]. These higher levels of differentiation are possible due to the capability of graphene-based materials to adsorb typical osteogenic inducers such as dexamethasone and *β*-glycerophosphate [33, 45]. Dexamethasone can be adsorbed due to *π*-*π* stacking between the aromatic rings in the molecules and the graphene basal plane [33]. GO is prone to bind to ascorbic acid due to the degree of hydrogen bonding that is formed between the OH moieties of the acid and GO [33, 45]. Hence, graphene and its derivatives allow the loading and release of drugs and proteins that can enhance the osteogenic differentiation of stem cells.

## 5. Combining Graphene and Various Materials to Enhance Osteogenic Differentiation

**Figure 6:**
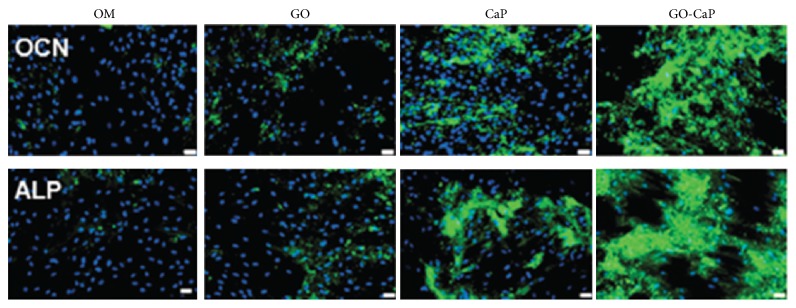
Immunofluorescence for osteocalcin and alkaline phosphatase (green) and DAPI (blue) after incubation with control osteogenic medium on GO, calcium phosphate (CaP), and GO–CaP for two weeks. Scale bars represent 20 mm. Reprinted with permission from [73] (Copyright (2014) American Chemical Society).

Although graphene has great benefits for osteogenic differentiations due its excellent physical properties, it can also be chemically modified [71] or combined with other materials like polymers, ceramics, and metals to further improve the differentiative potential [21, 62, 72–75]. GO is a widely used form of graphene due to the presence of carboxylic, epoxy, and hydroxide groups, which allow wide range of reactions and functionalization opportunities [12, 32, 66, 76]. Ceramic/functionalized graphene composites can improve biological outcomes of ceramic-based materials [72, 73, 75]. Hydroxyapatite (HA), for example, is a calcium phosphate ceramic commonly used for bone repair or regeneration due to its chemical similarity to that of natural apatite in bones [18]. The addition of graphene nanoplatelets (GNP) to 45S5 Bioglass results in a composite with high electrical conductivity and increased concentration of GNP. The electrically conductive biomaterials can be used in bone tissue engineering to facilitate cell growth and tissue regeneration with physioelectrical signal transfer [75]. The addition of GO to HA coatings can increase the coating adhesion strength on titanium sheets. The GO/HA composite coating also exhibits higher corrosion resistance than pure HA coatings. Furthermore, the GO-modified coating presents higher cell viability in comparison with titanium substrate regardless of the coating of HA [77]. By embedding graphene plates to calcium silicate matrix it was possible to improve the wear resistance of the composite created in a quantity-dependent manner. Although the addition of graphene to the calcium silicate does not increase osteoblastic-related gene expression of MSC, cell adhesion was enhanced by adding 1.5% of graphene to the material as compared to the calcium silicate alone [72]. The incorporation of GO to ultrathin plate shaped calcium phosphate nanoparticles improved the osteogenic differentiation of MSC (Figure 6). GO acts synergistically with calcium phosphate increasing calcium deposition, ALP activity, and OCN expression of MSC [73].

Polymers have also been modified with graphene to provide better environments for cell survival and differentiation. The addition of 1 wt% of GO to gelatin-based composite improves significantly the tensile strength, Young's modulus, and energy at break by 84, 65, and 158%, respectively [78]. The addition of rGO to chitosan changes its nanotopography due to the increase in roughness and surface area, thus enhancing adhesion and osteoblastic differentiation of MSCs. Due to the nanoscale disorder of graphene incorporated into the chitosan substrata, the mineralized deposits observed were higher as compared to chitosan and polystyrene substrate regardless of the use of osteogenic or culture media [21]. GO also increased the bioactivity of PCL during biomineralization by promoting the nucleation of HA nanoparticles in simulated body fluid [79].

Poly-L-lactide (PLLA) scaffold was modified with CNT and graphene of 1–3% wt by thermal-induced phase separation technique. The scaffold containing 3% wt of graphene enhances the differentiation of BMSC and increases the calcium deposition and formation of collagen type I. This can be attributed to the increase in specific surface area of the scaffold and the surface roughness that can increase adsorption of proteins. Furthermore, graphene provides more contacting surface to cells as compared to the same content of CNT [80].

Graphene can form self-supporting graphene hydrogel (SGH) by the principal of colloidal chemistry due to intrinsic corrugation of graphene and solvation repulsion between neighboring graphene sheets, resulting in a large amount of separated graphene sheets in a collective manner inside SGH [66, 81]. Multilayered SGH film allows the same level of cell adhesion and proliferation of BMSC in comparison to glass. The implantation of SGH film into subcutaneous sites of rats leads to formation of new blood vessels with minimal fibrous capsule formation after 12 weeks. Interestingly, this biocompatible film was able to stimulate osteogenic differentiation of stem cells without additional chemical inducers. This ability can be attributed to the corrugated and porous surface of the film that acts as anchor points for the cytoskeletons and exerts influence on cytoskeletal tension changing cell morphology [66]. A graphene-modified hydrogel prepared by hydrothermal method also contributes to a biocompatible three-dimensional environment as MG63 cells were capable of flourishing in the hydrogel for seven days. Guided filopodia protrusions of MG63 cell revealed that the cells were well adapted to the graphene hydrogel substrate [82].

Due to the large surface area and delocalized electrons, GO and rGO have the potential to bind and solubilize molecules acting as drug delivery vehicles [12, 33, 35, 83, 84]. Poly(l-lysine-graft-ethylene glycol)- (PLL-g-PEG-) coated PEDOT electrodes can be used as electroactive device for spatial-temporal controlled drug-release. Such devices can be used for long-term cell culturing and controlled differentiation of MSC through electrical stimulation [83].

Given that the induction of osteogenic differentiation of stem cells can take several weeks, the sustained release of inducing proteins, such as BMP-2, can accelerate this process. Recently the potential of titanium coated with GO (Ti/GO) has been explored for sustained release of BMP-2 to increase osteogenic differentiation* in vitro* and* in vivo* [63]. Ti substrates coated with GO enable loading and sustained release of BMP-2 without compromising the protein bioactivity. The* in vitro* osteogenic differentiation of BMSC was higher on Ti/GO combined with BMP-2 than on Ti with BMP-2. Further* in vivo* experiment demonstrated the efficacy of BMP-2 delivered by Ti or Ti/GO substrate after implantation into mouse calvaria defects. After 8 weeks, Ti/GO implants conjugated with BMP-2 showed extensive bone formation revealed by microcomputed tomography and histological analysis as compared to Ti/BMP-2 substrate [84].

These findings corroborate graphene as a promising material that can increment bioactivity and differentiative potential of candidate materials for bone tissue regeneration.

## 6. Conclusion

The characteristics of graphene such as large surface area, excellent mechanical properties, and feasibility to be transferred to different substrates among others make it a unique material for stem cell research. Graphene-modified substrates and materials are biocompatible, allow cell adhesion and proliferation, and increase differentiation of stem cells into osteogenic lineage. In addition, it can be easily functionalized to bind biomolecules or elements of choice to induce and control stem cell behavior. Although some challenges remain, the advances obtained by using graphene to induce osteogenesis are exciting. One of these challenges is the lack of thorough and profound understanding of the mechanism and signaling pathways involved in stem cell differentiation stimulated by graphene. Further studies at cellular and subcellular level beyond proof of concept and focusing on underlying mechanism are necessary. Moreover, comparisons of graphene with current known and successful biomaterial and implants must be performed to conclude the benefits of the material. Furthermore,* in vivo* animal studies are needed to assess biodistribution and their metabolic pathways in tissues and organs to permit future clinical applications.

Due to the unique structures and remarkable properties, graphene and its derivatives hold great potential for biomedical applications. Although the research with graphene for bone tissue regeneration is still in early stages of development, the material may have bright future in clinical scenarios.

## Figures and Tables

**Table 1:** Summary of studies using graphene for osteogenesis.

Material	Analysis	Outcomes	Reference
rGO-Chitosan	SEM, Alizarin Red staining, and immunofluorescence	The differentiation on rGO-chitosan substrate was higher than the ones obtained on the chitosan substrata and polystyrene regardless of the use of osteogenic induction media	[21]

rGO-PEDOT	Immunofluorescence staining, Alizarin Red S staining	The multifunctional rGO-PEDOT bioelectronic interface was used for manipulating attachment and orientation of MSC. The device acted as a drug releasing model under electrical modulation	[83]

GO	Immunofluorescence, microcomputed tomography, and Goldner trichrome	The osteogenetic differentiation of human BMMSCs on Ti/GO substrate was higher compared to Ti substrate	[84]

GONR, rGONR	Immunofluorescence staining and Alizarin Red staining	Graphene nanogrids increase the osteogenic differentiation of BMSC; the differentiation coincides with the patterns of the nanogrids	[45]

CVD	Immunofluorescence staining	The cells adhered and proliferated more on CVD-grown graphene than on SiO_2_ substrates	[46]

CVD, GO	Immunofluorescence staining and Alizarin Red staining	Graphene was capable of preconcentrating osteogenic differentiation factors. GO strongly enhances adipogenic differentiation	[33]

CVD	Cell viability assay, immunofluorescence staining, and Alizarin Red staining	CVD-grown graphene allowed the proliferation of MSC and increased the differentiation towards osteoblast	[47]

3DGp	Immunofluorescence staining and SEM	3DGp maintains MSC viability and promotes osteogenic differentiation without the use of chemical inducers	[52]

CaS-G	MTT, SEM, and RT-PCR	Cell adhesion was enhanced by adding 1.5% of graphene to the material as compared to the calcium silicate alone	[72]

SGH	MTT, H & E, immunofluorescence staining, and Alizarin staining	The self-supporting graphene hydrogel (SGH) film allows cell adhesion and proliferation and accelerates the osteogenic differentiation without chemical inducer	[66]

GO-CaP	Alizarin Red S staining RT PCR and immunofluorescence	The GO-CaP nanocomposite exhibited superior osteoinductivity compared to individual or combined effects of GO and CaP	[73]

Carbon nanotubes and graphene	SEM, Elisa, and H & E staining	Cells in PLLA composite scaffolds containing 3% wt of graphene presented higher expression of osteogenesis-related proteins, calcium deposition, and the formation of type I collagen	[80]

Graphene hydrogel	MTT and SEM	Graphene 3D hydrogel allows cell proliferation and attachment confirming the biocompatibility of the graphene hydrogel scaffolds	[82]

## References

[1] Rose F. R. A. J., Oreffo R. O. C. (2002). Bone tissue engineering: hope vs hype. *Biochemical and Biophysical Research Communications*.

[2] Bae H., Chu H., Edalat F. (2014). Development of functional biomaterials with micro- and nanoscale technologies for tissue engineering and drug delivery applications. *Journal of Tissue Engineering and Regenerative Medicine*.

[3] Rosa V., Della Bona A., Cavalcanti B. N., Nör J. E. (2012). Tissue engineering: from research to dental clinics. *Dental Materials*.

[4] Lutolf M. P. (2009). Biomaterials: spotlight on hydrogels. *Nature Materials*.

[5] Mizuno M., Shindo M., Kobayashi D., Tsuruga E., Amemiya A., Kuboki Y. (1997). Osteogenesis by bone marrow stromal cells maintained on type I collagen matrix gels in vivo. *Bone*.

[6] Rosa V., Zhang Z., Grande R. H. M., Nör J. E. (2013). Dental pulp tissue engineering in full-length human root canals. *Journal of Dental Research*.

[7] El-Fiqi A., Lee J. H., Lee E.-J., Kim H.-W. (2013). Collagen hydrogels incorporated with surface-aminated mesoporous nanobioactive glass: improvement of physicochemical stability and mechanical properties is effective for hard tissue engineering. *Acta Biomaterialia*.

[8] Lu Q., Pandya M., Jalil R. A. Modulation of dental pulp stem cell odontogenesis in a tunable PEG-fibrinogen hydrogel system.

[9] Salinas C. N., Anseth K. S. (2008). The enhancement of chondrogenic differentiation of human mesenchymal stem cells by enzymatically regulated RGD functionalities. *Biomaterials*.

[10] Lee C., Wei X., Kysar J. W., Hone J. (2008). Measurement of the elastic properties and intrinsic strength of monolayer graphene. *Science*.

[11] Khare R., Mielke S. L., Paci J. T. (2007). Coupled quantum mechanical/molecular mechanical modeling of the fracture of defective carbon nanotubes and graphene sheets. *Physical Review B*.

[12] Loh K. P., Bao Q., Ang P. K., Yang J. (2010). The chemistry of graphene. *Journal of Materials Chemistry*.

[13] Song Y. J., Wei W. L., Qu X. G. (2011). Colorimetric biosensing using smart materials. *Advanced Materials*.

[14] Feng L., Liu Z. (2011). Graphene in biomedicine: opportunities and challenges. *Nanomedicine*.

[15] Kostarelos K., Novoselov K. S. (2014). Materials science. Exploring the interface of graphene and biology. *Science*.

[16] Goenka S., Sant V., Sant S. (2014). Graphene-based nanomaterials for drug delivery and tissue engineering. *Journal of Controlled Release*.

[17] Liu J., Cui L., Losic D. (2013). Graphene and graphene oxide as new nanocarriers for drug delivery applications. *Acta Biomaterialia*.

[18] Depan D., Girase B., Shah J. S., Misra R. D. K. (2011). Structure-process-property relationship of the polar graphene oxide-mediated cellular response and stimulated growth of osteoblasts on hybrid chitosan network structure nanocomposite scaffolds. *Acta Biomaterialia*.

[19] Depan D., Misra R. D. K. (2013). The interplay between nanostructured carbon-grafted chitosan scaffolds and protein adsorption on the cellular response of osteoblasts: structure-function property relationship. *Acta Biomaterialia*.

[20] Kim J., Choi K. S., Kim Y. (2013). Bioactive effects of graphene oxide cell culture substratum on structure and function of human adipose-derived stem cells. *Journal of Biomedical Materials Research: Part A*.

[21] Kim J., Kim Y.-R., Kim Y. (2013). Graphene-incorporated chitosan substrata for adhesion and differentiation of human mesenchymal stem cells. *Journal of Materials Chemistry B*.

[22] Ryu S., Kim B.-S. (2013). Culture of neural cells and stem cells on graphene. *Tissue Engineering and Regenerative Medicine*.

[23] Chen G.-Y., Pang D. W.-P., Hwang S.-M., Tuan H.-Y., Hu Y.-C. (2012). A graphene-based platform for induced pluripotent stem cells culture and differentiation. *Biomaterials*.

[24] Geim A. K., Novoselov K. S. (2007). The rise of graphene. *Nature Materials*.

[25] Nair R. R., Blake P., Grigorenko A. N. (2008). Fine structure constant defines visual transparency of graphene. *Science*.

[26] Castro Neto A. H., Guinea F., Peres N. M. R., Novoselov K. S., Geim A. K. (2009). The electronic properties of graphene. *Reviews of Modern Physics*.

[27] Bunch J. S., Verbridge S. S., Alden J. S. (2008). Impermeable atomic membranes from graphene sheets. *Nano Letters*.

[28] Zhang L., Zhang F., Yang X. (2013). Porous 3D graphene-based bulk materials with exceptional high surface area and excellent conductivity for supercapacitors. *Scientific Reports*.

[29] Liu F., Ming P., Li J. (2007). *Ab initio* calculation of ideal strength and phonon instability of graphene under tension. *Physical Review B*.

[30] Novoselov K. S., Geim A. K., Morozov S. V. (2004). Electric field in atomically thin carbon films. *Science*.

[31] Ivanovskii A. L. (2012). Graphene-based and graphene-like materials. *Russian Chemical Reviews*.

[32] Pei S. F., Cheng H.-M. (2012). The reduction of graphene oxide. *Carbon*.

[33] Lee W. C., Lim C. H. Y. X., Shi H. (2011). Origin of enhanced stem cell growth and differentiation on graphene and graphene oxide. *ACS Nano*.

[34] Chung C., Kim Y.-K., Shin D., Ryoo S.-R., Hong B. H., Min D.-H. (2013). Biomedical applications of graphene and graphene oxide. *Accounts of Chemical Research*.

[35] Dreyer D. R., Park S., Bielawski C. W., Ruoff R. S. (2010). The chemistry of graphene oxide. *Chemical Society Reviews*.

[36] Rodríguez-Lozano F. J., García-Bernal D., Aznar-Cervantes S. (2014). Effects of composite films of silk fibroin and graphene oxide on the proliferation, cell viability and mesenchymal phenotype of periodontal ligament stem cells. *Journal of Materials Science: Materials in Medicine*.

[37] Elkhenany H., Amelse L., Lafont A. (2015). Graphene supports *in vitro* proliferation and osteogenic differentiation of goat adult mesenchymal stem cells: potential for bone tissue engineering. *Journal of Applied Toxicology*.

[38] Kim S.-M., Kim J.-H., Kim K.-S. (2014). Synthesis of CVD-graphene on rapidly heated copper foils. *Nanoscale*.

[39] Casiraghi C., Hartschuh A., Lidorikis E. (2007). Rayleigh imaging of graphene and graphene layers. *Nano Letters*.

[40] Georgakilas V., Otyepka M., Bourlinos A. B. (2012). Functionalization of graphene: covalent and non-covalent approaches, derivatives and applications. *Chemical Reviews*.

[41] Park S., Ruoff R. S. (2009). Chemical methods for the production of graphenes. *Nature Nanotechnology*.

[42] Li X., Cai W., An J. (2009). Large-area synthesis of high-quality and uniform graphene films on copper foils. *Science*.

[43] Ryoo S.-R., Kim Y.-K., Kim M.-H., Min D.-H. (2010). Behaviors of NIH-3T3 fibroblasts on graphene/carbon nanotubes: proliferation, focal adhesion, and gene transfection studies. *ACS Nano*.

[44] Akhavan O., Ghaderi E. (2013). Differentiation of human neural stem cells into neural networks on graphene nanogrids. *Journal of Materials Chemistry B*.

[45] Akhavan O., Ghaderi E., Shahsavar M. (2013). Graphene nanogrids for selective and fast osteogenic differentiation of human mesenchymal stem cells. *Carbon*.

[46] Kalbacova M., Broz A., Kong J., Kalbac M. (2010). Graphene substrates promote adherence of human osteoblasts and mesenchymal stromal cells. *Carbon*.

[47] Nayak T. R., Andersen H., Makam V. S. (2011). Graphene for controlled and accelerated osteogenic differentiation of human mesenchymal stem cells. *ACS Nano*.

[48] Ferrari A. C., Meyer J. C., Scardaci V. (2006). Raman spectrum of graphene and graphene layers. *Physical Review Letters*.

[49] Zhang Y., Ali S. F., Dervishi E. (2010). Cytotoxicity effects of graphene and single-wall carbon nanotubes in neural phaeochromocytoma-derived pc12 cells. *ACS Nano*.

[50] Akhavan O., Kalaee M., Alavi Z. S., Ghiasi S. M. A., Esfandiar A. (2012). Increasing the antioxidant activity of green tea polyphenols in the presence of iron for the reduction of graphene oxide. *Carbon*.

[51] Naujoks C., Langenbach F., Berr K. (2011). Biocompatibility of osteogenic predifferentiated human cord blood stem cells with biomaterials and the influence of the biomaterial on the process of differentiation. *Journal of Biomaterials Applications*.

[52] Crowder S. W., Prasai D., Rath R. (2013). Three-dimensional graphene foams promote osteogenic differentiation of human mesenchymal stem cells. *Nanoscale*.

[53] Sasidharan A., Panchakarla L. S., Chandran P. (2011). Differential nano-bio interactions and toxicity effects of pristine versus functionalized graphene. *Nanoscale*.

[54] Li Y., Yuan H., von dem Bussche A. (2013). Graphene microsheets enter cells through spontaneous membrane penetration at edge asperities and corner sites. *Proceedings of the National Academy of Sciences of the United States of America*.

[55] Yang K., Wan J., Zhang S., Zhang Y., Lee S.-T., Liu Z. (2011). In vivo pharmacokinetics, long-term biodistribution, and toxicology of pegylated graphene in mice. *ACS Nano*.

[56] Singh S. K., Singh M. K., Nayak M. K. (2011). Thrombus inducing property of atomically thin graphene oxide sheets. *ACS Nano*.

[57] Dawson J. I., Oreffo R. O. C. (2008). Bridging the regeneration gap: stem cells, biomaterials and clinical translation in bone tissue engineering. *Archives of Biochemistry and Biophysics*.

[58] Willerth S. M., Sakiyama-Elbert S. E. (2008). Combining stem cells and biomaterial scaffolds for constructing tissues and cell delivery. *StemBook*.

[59] Liu X., Ma P. X. (2004). Polymeric scaffolds for bone tissue engineering. *Annals of Biomedical Engineering*.

[60] Rezwan K., Chen Q. Z., Blaker J. J., Boccaccini A. R. (2006). Biodegradable and bioactive porous polymer/inorganic composite scaffolds for bone tissue engineering. *Biomaterials*.

[61] Mooney E., Dockery P., Greiser U., Murphy M., Barron V. (2008). Carbon nanotubes and mesenchymal stem cells: biocompatibility, proliferation and differentiation. *Nano Letters*.

[62] Qi W. Y., Yuan W., Yan J., Wang H. (2014). Growth and accelerated differentiation of mesenchymal stem cells on graphene oxide/poly-L-lysine composite films. *Journal of Materials Chemistry B*.

[63] La W. G., Kang S. W., Yang H. S. (2010). The efficacy of bone morphogenetic protein-2 depends on its mode of delivery. *Artificial Organs*.

[64] Li N., Zhang Q., Gao S. (2013). Three-dimensional graphene foam as a biocompatible and conductive scaffold for neural stem cells. *Scientific Reports*.

[65] McBeath R., Pirone D. M., Nelson C. M., Bhadriraju K., Chen C. S. (2004). Cell shape, cytoskeletal tension, and RhoA regulate stem cell lineage commitment. *Developmental Cell*.

[66] Lu J., He Y.-S., Cheng C. (2013). Self-supporting graphene hydrogel film as an experimental platform to evaluate the potential of graphene for bone regeneration. *Advanced Functional Materials*.

[67] Ryoo H. M., Hoffmann H. M., Beumer T. (1997). Stage-specific expression of Dlx-5 during osteoblast differentiation: involvement in regulation of osteocalcin gene expression. *Molecular Endocrinology*.

[68] Guilak F., Cohen D. M., Estes B. T., Gimble J. M., Liedtke W., Chen C. S. (2009). Control of stem cell fate by physical interactions with the extracellular matrix. *Cell Stem Cell*.

[69] Higuchi A., Ling Q.-D., Chang Y., Hsu S.-T., Umezawa A. (2013). Physical cues of biomaterials guide stem cell differentiation fate. *Chemical Reviews*.

[70] Habibovic P., Yuan H., van der Valk C. M., Meijer G., van Blitterswijk C. A., de Groot K. (2005). 3D microenvironment as essential element for osteoinduction by biomaterials. *Biomaterials*.

[71] Tang L. A. L., Lee W. C., Shi H. (2012). Highly wrinkled cross-linked graphene oxide membranes for biological and charge-storage applications. *Small*.

[72] Xie Y., Li H., Zhang C., Gu X., Zheng X., Huang L. (2014). Graphene-reinforced calcium silicate coatings for load-bearing implants. *Biomedical Materials*.

[73] Tatavarty R., Ding H., Lu G., Taylor R. J., Bi X. (2014). Synergistic acceleration in the osteogenesis of human mesenchymal stem cells by graphene oxide–calcium phosphate nanocomposites. *Chemical Communications*.

[74] Gao C., Liu T., Shuai C., Peng S. (2014). Enhancement mechanisms of graphene in nano-58S bioactive glass scaffold: mechanical and biological performance. *Scientific Reports*.

[75] Porwal H., Grasso S., Cordero-Arias L., Li C., Boccaccini A. R., Reece M. J. (2014). Processing and bioactivity of 45S5 bioglass-graphene nanoplatelets composites. *Journal of Materials Science: Materials in Medicine*.

[76] Kang S.-W., La W.-G., Kang J. M., Park J.-H., Kim B.-S. (2008). Bone morphogenetic protein-2 enhances bone regeneration mediated by transplantation of osteogenically undifferentiated bone marrow-derived mesenchymal stem cells. *Biotechnology Letters*.

[77] Li M., Liu Q., Jia Z. (2014). Graphene oxide/hydroxyapatite composite coatings fabricated by electrophoretic nanotechnology for biological applications. *Carbon*.

[78] Wan C., Frydrych M., Chen B. (2011). Strong and bioactive gelatin-graphene oxide nanocomposites. *Soft Matter*.

[79] Wan C., Chen B. (2011). Poly(*ε*-caprolactone)/graphene oxide biocomposites: mechanical properties and bioactivity. *Biomedical Materials*.

[80] Duan S., Yang X., Mei F. (2015). Enhanced osteogenic differentiation of mesenchymal stem cells on poly(l-lactide) nanofibrous scaffolds containing carbon nanomaterials. *Journal of Biomedical Materials Research Part A*.

[81] Cheng C., Li D. (2013). Solvated graphenes: an emerging class of functional soft materials. *Advanced Materials*.

[82] Lim H. N., Huang N. M., Lim S. S., Harrison I., Chia C. H. (2011). Fabrication and characterization of graphene hydrogel via hydrothermal approach as a scaffold for preliminary study of cell growth. *International Journal of Nanomedicine*.

[83] Hsiao Y.-S., Kuo C.-W., Chen P. (2013). Multifunctional graphene-pedot microelectrodes for on-chip manipulation of human mesenchymal stem cells. *Advanced Functional Materials*.

[84] La W. G., Park S., Yoon H. H. (2013). Delivery of a therapeutic protein for bone regeneration from a substrate coated with graphene oxide. *Small*.

